# Comparing proton treatment plans of pediatric brain tumors in two pencil beam scanning nozzles with different spot sizes

**DOI:** 10.1120/jacmp.v16i6.5389

**Published:** 2015-11-08

**Authors:** John C. Kralik, Liwen Xi, Timothy D. Solberg, Charles B. Simone, Liyong Lin

**Affiliations:** ^1^ Department of Radiation Oncology University of Pennsylvania Philadelphia PA USA; ^2^ Biometrics and Reporting Janssen Research & Development, LLC Spring House PA USA

**Keywords:** treatment planning, pencil beam scanning, pediatric, brain

## Abstract

Target coverage and organ‐at‐risk sparing were compared for 22 pediatric patients with primary brain tumors treated using two distinct nozzles in pencil beam scanning (PBS) proton therapy. Consecutive patients treated at our institution using a PBS‐dedicated nozzle (DN) were replanned using a universal nozzle (UN) beam model and the original DN plan objectives. Various cranial sites were treated among the patients to prescription doses ranging from 45 to 54 Gy. Organs at risk (OARs) evaluated were patient‐dependent; 15 unique OARs were analyzed, all of which were assessed in at least 10 patients. Clinical target volume (CTV) coverage and organ sparing were compared for the two nozzles using dose‐volume histogram data. Statistical analysis using a confidence‐interval approach demonstrates that CTV coverage is equivalent for UN and DN plans within ±5% equivalence bounds. In contrast, average mean and maximum doses are significantly higher for nearly all 15 OARs in the UN plans. The average median increase over all OARs and patients is approximately 1.7 Gy, with an increase in the 25%–75% of 1.0–2.3 Gy; the median increase to the pituitary gland, temporal lobes, eyes and cochleas are 1.8, 1.7, 0.7, and 2.7 Gy, respectively. The CTV dose distributions fall off slower for UN than for the DN plans; hence, normal tissue structures in close proximity to CTVs receive higher doses in UN plans than in DN plans. The higher OAR doses in the UN plans are likely due to the larger spot profile in plans created with UN beams. In light of the high rates of toxicities in pediatric patients receiving cranial irradiation and in light of selected brain tumor types having high cure rates, this study suggests the smaller DN beam profile is preferable for the advantage of reducing dose to OARs.

PACS number: 87.55.D‐

## I. INTRODUCTION

Retrospective planning can be used to evaluate dosimetric differences between proton‐ and photon‐based modalities. Prior studies have demonstrated a dosimetric advantage when delivering proton therapy compared with intensity‐modulated radiation therapy (IMRT) for a variety of malignancies, including pediatric parameningeal rhabdomyosarcomas,^(1)^ head and neck cancer,^(2)^ seminoma,^(3)^ and gastric tumors.^(4)^ More recently, comparisons have been made between pencil beam scanning and passive scattering proton modalities.^5^, ^6^ This study represents a retrospective dosimetric evaluation of patients with pediatric brain tumors using pencil beam scanning beam lines that utilize one of two distinct treatment nozzles. A Monte Carlo simulation by Hyer et al.^(7)^ demonstrated that a smaller proton spot can reduce field penumbra between the treatment target and adjacent critical organs. The same group demonstrated experimentally that a reduction in the spot size/lateral dose penumbra via a novel dynamic collimating system maintains coverage while significantly decreasing normal tissue exposure, analogous to treatments using a smaller proton beam spot size.^(8)^

**Figure 1:**
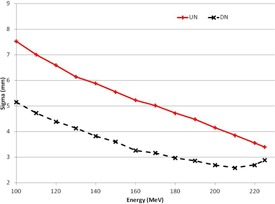
One‐sigma spot profiles for DN (black, dashed) and UN (red, solid). The one‐sigma beam size measured at isocenter is shown as a function of beam energy.

Our center delivers pencil beam scanning (PBS) proton therapy using two different nozzles: a dedicated nozzle (DN) commissioned in 2012, and a universal nozzle (UN) commissioned in 2013. Both are manufactured by IBA (Ion Beam Applications SA, Louvain‐la‐Neuve, Belgium). The UN is designed to deliver pencil beam, uniform scanning, and double scattered beams, while the DN is designed only for pencil beam delivery.^(9)^ The beam profiles from each nozzle were measured and described previously.^9^, ^10^, ^11^, ^12^ Each beam profile consists of a central Gaussian profile surrounded by a low‐dose halo that extends beyond the central Gaussian. A plot of the one‐sigma spot size at isocenter as a function of beam energy is shown in Fig. 1 for each of the nozzles. The UN spot is at least 1 mm larger than the DN spot at isocenter for beam energies of 100–210 MeV. As the DN and the UN share the same energy selection system, and beamline components within the treatment nozzles consist of only MU chambers and thin vacuum windows, the shape of the two Bragg peaks is indistinguishable. Both DN and UN are commissioned using the same measurement technique and for the same proton dose algorithm (convolution superposition version 10.0.28). Spot profiles are measured using a 0.5 mm resolution scintillation detector and approximated by two‐dimensional Gaussian models; the Bragg peaks are collected using an 80 mm diameter parallel chamber.^8^, ^9^, ^11^, ^12^

Existing data assessing how doses to target volumes and organs at risk (OARs) differ according to different nozzles and spot profiles are sparse. The aim of this study is to compare the target coverage and OAR sparing of plans calculated using DN and UN via retrospective treatment planning in pediatric brain tumor patients. We hypothesize that compared with plans using UN, plans using DN could allow for a more conformal treatment and reduce doses to OARs overlapping with or in immediate proximity to the target volume being treated. Given the deployment and use of the UN, as well as the imminent commissioning of new scanning proton beam facilities, it is useful to examine whether the dosimetric differences between the DN and UN nozzle systems may influence the choice of nozzle, or decisions to retrofit UNs with a dynamic aperture.

## II. MATERIALS AND METHODS

The plans of 22 consecutive pediatric brain tumor patients who were treated at a single institution using the DN were replanned using the UN beam model. This cohort of 11 males and 11 females was treated in 2012 and 2013. Patients ranged in age from 4 to 23 years old at the time of treatment, with a median age of 12.5 years. The treatment site varied among the group, with nine patients having right‐sided tumors, five having left‐sided tumors, and the rest having tumors located along the midline. A summary of patient characteristics is provided in Table 1.

Note in Table 1 that the plans for three patients^8^, ^9^, ^13^ originally included partial treatment using intensity modulated radiation therapy (IMRT). The PBS portion of two of those patients^9^, ^13^ was renormalized to 54 Gy for the UN/DN comparison. In addition, the PBS boost treatments for three of the patients^14^, ^15^, ^16^ were not included in the UN/DN comparison as a matter of convenience, and because the boost doses were substantially lower than the initial treatment plans. In contrast, it was necessary to sum both initial and boost components of the plans for two patients^11^, ^17^ because both comprised approximately 50% of the prescribed dose.

All treatment plans examined in this study were created using Varian Eclipse External Beam Planning version 11.0 (Varian Medical Systems, Palo Alto, CA).^(14)^ Each plan consisted of two coronal fields, delivered by a horizontal fixed beam (DN) or a fixed gantry angle of 90° (UN) to ensure a fair planning comparison between the two nozzles. The two‐field arrangement maximizes plan quality while minimizing the treatment time for this pediatric cohort. Each plan was optimized using a single‐field uniform dose technique and the dose was calculated on a 0.25 cm grid. The spacing between beam spots is specified in the Eclipse beam configuration to be proportional to the minimal spot size at the patient entrance; typically this is 0.5–0.6 cm when using the DN, and 0.6–0.8 cm for the UN plans. Such a choice of spot spacing reduces the number of spots in treatment plans without large plan quality degradation. The layer spacing was chosen to be four times the range sigma so that a reasonable number of layers are used while still maintaining dose homogeneity within target along the beam direction. Note too, that a universal bolus was used rather than a range shifter in both nozzles; this reduces spot size at depths that are shallower than 75 mm.^(15)^

**Table 1:** Patient characteristics for the current study. Here, PBS refers to pencil beam (proton) therapy; US refers to uniform scanning proton therapy; IMRT refers to intensity‐modulated (X‐ray) radiation therapy; DS refers to double scattering proton therapy; and 3D CRT refers to 3D conformal (X‐ray) radiation therapy

*Patient No.*	*Diagnosis*	*Target Volume(s)*	*Prescription Dose (Modality)*
1	Optic glioma	21.4 cc	54 Gy (PBS)
2	Anaplastic ependymoma, WHO grade III	7.2 cc	54 Gy (PBS)
3	Recurrent standard risk medulloblastoma (reirradiation)	5.8 cc	45 Gy (PBS)+9 Gy (US)
4	Astrocytoma, grade II	45.5 cc	54 Gy (PBS)
5	Recurrent right acoustic neuroma (reirradiation)	20.3 cc	46.8 Gy (PBS)
6	Extraventricular neurocytoma, WHO grade II	47.4 cc	54 Gy (PBS)
7	Pituitary macroadenoma, secreting	96.9 cc	52.2 Gy (PBS)
8	Astrocytoma, grade III	112.7 cc	50.4 Gy (PBS)+9 Gy (IMRT)
9	Cavernous sinus melanoma	33.0 cc	32.4 Gy^a^ (PBS) + 34.2 Gy (IMRT)
10	Adamantinomatous craniopharyngioma	8.4 cc	54 Gy (PBS)
11	CNS germinoma	233.3/42.9 cc	23.4 Gy (PBS, initial)+21.6 Gy (PBS boost)
12	Supratentorial ependymoma, grade II	88.5 cc	54 Gy (PBS)+5.4 Gy (DS)
13	Glioblastoma multiforme, WHO grade IV	249.2 cc	46 Gy^b^ (PBS) + 14 Gy (PBS)
14	Recurrent grade II ependymoma (reirradiation)	50.4 cc	50.4 Gy^b^ (PBS, initial) + 9 Gy (PBS, boost)
15	Recurrent medulloblastoma (reirradiation)	59.3 cc	54 Gy (PBS)
16	Atypical teratoid rhabdoid tumor	159.9 cc	54 Gy (PBS)
17	Pilocytic astrocytoma	26.4 cc	54 Gy (PBS)
18	Anaplastic astrocytoma, WHO grade III	131 cc	45 Gy^b^ (PBS, initial) + 14.4 Gy (PBS, boost)
19	High‐risk medulloblastoma (M2 disease)	165.4 cc	19.8 Gy^a^ (PBS, Brain) + 9 Gy (Brain, 3DCRT) + 9 Gy (DS, Spine)
20	Germinoma of the pituitary stalk	2.3/2.3 cc	23.4 Gy (PBS, initial)+21.6 Gy (PBS, boost)
21	Thalamic astrocytoma, grade II	112.1 cc	54 Gy (PBS)
22	Thalamic astrocytoma, grade II	33.7 cc	54 Gy (PBS)

a Renormalized to 54 Gy for UN, DN comparison.

b Used only the initial PBS plans for the UN, DN comparisons.

The PTVs were uniformly expanded 5 mm from the CTVs and were used for evaluation purposes only. Adequate lateral coverage of the CTV was achieved using one additional scan spot around the field perimeter. Different distal and proximal margins for each field arise mainly from the range uncertainties of CT images (∼3% of range plus 1–3 mm, depending on the proximity of adjacent OARs), and the same margins were used for UN and DN plans. One planning target volume is created to best accommodate different beam specific planning target volumes^(18)^ at each treatment field for single‐field optimization (SFO); the same planning target is used for both DN and UN plans.

The optimization objectives for plans created for the UN were copied from the PBS plans from the DN. Thus, the constraint type, number of constraints per structure, volume and dose targets per structure, and weighting were preserved in the new and fully optimized UN plans. Following full optimization, the new plans were normalized to achieve the same $D_{95\%}$ to the CTV as the original plans, thereby preserving $D_{\text{mean}}$ to the CTVs. Of note, all of the cases replanned on the UN produced clinically acceptable plans.

Dose analysis for OARs was performed using dose‐volume histograms (DVHs). In order to limit exposure of the analysis to spurious dose levels to the OAR, dose values were recorded only when values exceeded 50 cGy for either the DN or UN plans.

Equivalence between the UN and the DN plans in prescription dose‐normalized mean, maximum, and minimum CTV doses was assessed using a confidence interval approach with ±5% equivalence bounds. Percent differences between the UN and DN plans in prescription dose‐normalized mean, maximum, and minimum CTV doses were calculated. The means of these percent differences and the associated 90% bootstrap confidence intervals were computed.^(19)^ Associations between mean dose differences to the OARs between the UN and the DN plans versus the age, tumor location, CTV size, and OAR size were examined using Spearman's rank correlation rho.^(20)^

CTV coverage was quantified by the mean, maximum, and minimum dose values in the CTV normalized to the prescription dose $D_{\text{Rx}}$, or $D_{\text{mean}}/D_{\text{Rx}}$, $D_{\text{max}}/D_{\text{Rx}}$, and $D_{\text{min}}/D_{\text{Rx}}$, respectively. Additional metrics included the relative CTV volume receiving at least 95% of the prescription dose, $V_{95\%}$, and the normalized dose to 5% and 95% of the CTV, $D_{5\%}$ and $D_{95\%}$, respectively.

CTV coverage in UN and DN plans was assessed using a confidence interval (CI) approach with equivalence bounds set to ± 5%. The 90% CIs on the mean percent change in various CTV dose metrics from UN and DN plans were calculated using a bootstrap method.^(18)^ Specifically, the percent changes, $(D({\text{UN})}_{i}-D({\text{DN})}_{i})/D({\text{DN})}_{i}$, for mean, maximum, and minimum CTV doses and for the normalized volume coverage, $(V({\text{UN})}_{95\%}-V({\text{DN})}_{95\%})/V({\text{DN})}_{95\%}$, were calculated.

Finally, a robustness study^21^, ^22^ was conducted on all plans, comprising isocenter shifts of ± 2 mm along each coordinate axis along with CT Hounsfield unit excursions of ± 2.5%. This led to the creation of 12 DVHs for each CTV and OAR. CTV coverage and OAR doses were compared for UN and DN plans.

## III. RESULTS

Table 2 summarizes the comparison of CTV coverage in UN and DN plans. The 90% CIs for all of the metrics lie within the equivalence bounds, indicating that in terms of CTV coverage, the UN plans are equivalent to DN plans.

The CTV coverage quality was also quantified using the homogeneity index (HI) and the inhomogeneity coefficient (IC).^(5)^ These metrics, which are derived from the quantities described above, are given by

(1) $$\text{HI}=(D_{\text{max}}-D_{\text{min}})/D_{\text{Rx}}$$

and

(2) $$\text{IC}=(D_{5\%}-D_{95\%})/D_{\text{mean}}$$

When the CTV coverage is optimized, both HI and IC tend toward zero. The average HI for the UN and DN plans was found to be similar, at 0.19±0.12 and 0.17±0.12, respectively, though not statistically equivalent (CI=(17%, 46%)). Likewise, the IC was found to be similar at 0.046±0.024 for the UN and 0.032±0.026 for the DN, but again, not statistically equivalent (CI=(40%, 73%)). Further differences in HI and IC are addressed in the discussion section below.

**Figure 2:**
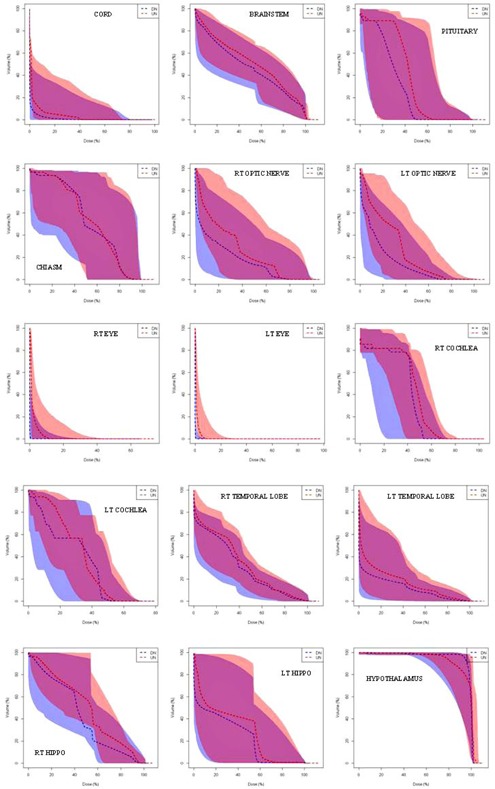
Cumulative OAR DVHs taken from UN (red) and DN (blue) plans. Laterality is denoted LT and RT for left and right, respectively, and HIPPO denotes the hippocampus. Relative doses are normalized to the prescription doses. The dashed lines represent median values among patients and the shaded range represents the 25%–75% interquartile range.

A comparison of cumulative DVHs for 12 of the OARs is shown in Fig. 2. These DVH graphs, which were created using the R‐package, RadOnc,^(16)^ display the interquartile (25%–75%) ranges among patients from UN and DN plans. In these plots, the shaded regions representing the UN (red shaded) and DN (blue shaded) plans overlap; however, the UN DVH ranges are generally wider and extend to higher doses than the DN DVH ranges. Likewise, the median DVH data, indicated by the dashed lines in the graphs, are generally higher for the UN plans than for the DN plans. For example, the $D_{50\%}$ for the median pituitary gland dose is about 50% higher in UN plans, and the cumulative doses to the right and left eyes are more than twice higher in UN plans. The median doses to the optic nerves are 10%–100% higher at nearly all volumes in UN plans, while the right and left temporal lobes receive about 5%–20% more dose in the UN plans. Also, the lower bound for the second quartile for the cochleas is about twice as large for the UN plans at nearly all relative volumes.

Doses to OARs derived from the DVHs are presented in Table 3, which lists mean, maximum, and standard deviation of doses to the 15 unique OARs that were clinically assessed. As the treatment site varied among the patients, all 15 OARs were not quantified for all 22 patients; however, all were assessed in at least 10 patients. The number of patients with a given OAR measurement is listed in the second column of the table.

**Figure 3:**
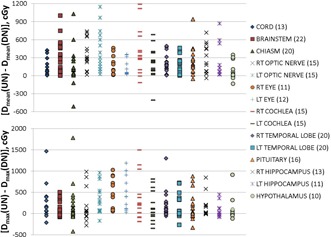
Individual difference in mean (upper plot) and maximum (lower plot) dose to OARs. $D_{\text{mean}}$ (UN, DN) = mean dose to OAR on the UN, DN, respectively. $D_{\text{max}}$ (UN or DN) = max dose to OARs on the UN or DN. The numbers in parentheses next to the OAR labels are the number of patients which are included in the sample for that OAR.

The data in Table 3 demonstrate that both the mean and maximum OAR doses are higher for the UN plans. Figure 3 shows graphically the individual difference in mean and maximum doses, $D_{\text{mean}}(\text{UN})-D_{\text{mean}}(\text{DN})$, and $D_{\text{max}}(\text{UN})-D_{\text{max}}(\text{DN})$, respectively, to each of the individual 15 OARs. The number of patients, or sample size, for each OAR is listed in parentheses after the OAR label on the right‐hand side of the graph. As mentioned earlier, data were included for a given OAR only if either $D_{\text{mean}}$ or $D_{\text{max}}$ values were at least 50 cGy.

**Table 2:** CTV coverage statistics

*Metric*	*Dedicated Nozzle*	*Universal Nozzle*	*Normalized Change (90% CI)*
$D_{\text{mean}}/D_{\text{Rx}}$	1.05±0.04	1.05±0.04	CI=(0.30%, 0.62%)
$V_{95\%}$	98.8±3%	99.0±2%	CI=(−0.44%,−0.05%)
$D_{\text{max}}/D_{\text{Rx}}$	1.09±0.04	1.10±0.05	CI=(0.88%, 1.7%)
$D_{\text{min}}/D_{\text{Rx}}$	0.92±0.11	0.91±0.12	CI=(−4.5%,−0.72%)
$D_{5\%}/D_{\text{mean}}$	1.015±0.008	1.021±0.009	CI=(0.75%, 1.3%)
$D_{95\%}/D_{\text{mean}}$	0.979±0.021	0.971±0.020	CI=(−0.68%,−0.08%)

$V_{95\%}$ = minimum dose to 95% of the CTV volume; $D_{\text{max}},D_{\text{min}}$ = max., min. dose to the CTV; $D_{5\%},D_{95\%}$ = dose to 5%, 95% of the CTV volume; CI = confidence interval.

**Table 3:** OAR doses, where dose values are presented as means ± standard deviations

		*Dedicated Nozzle*		*Universal Nozzle*	
*OAR*	*No. Patients*	$D_{\text{mean}}\;(\text{cGy})$	$D_{\text{max}}\;(\text{cGy})$	$D_{\text{mean}}\;(\text{cGy})$	$D_{\text{max}}\;(\text{cGy})$
Spinal Cord	13	530±690	2180±2080	700±810	2450±2050
Brainstem	22	2480±1610	4830±1630	2780±1590	4920±1590
Optic Chiasm	20	2780±2030	3760±2240	2920±1970	3950±2000
R. Optic Nerve	15	1530±1770	3390±2140	1890±1760	3560±1970
L. Optic Nerve	15	1420±1740	3150±2060	1840±1720	3490±1850
R. Eye	11	65±85	910±1190	230±240	1400±1360
L. Eye	12	150±460	730±1680	280±550	1210±1660
R. Cochlea	15	2100±1390	2900±1650	2500±1270	3220±1390
L. Cochlea	15	1540±1160	2350±1520	1740±1110	2520±1320
R. Temporal Lobe	20	1530±1180	4200±1970	1710±1240	4380±1800
L. Temporal Lobe	20	1110±1240	4040±2150	1270±1300	4200±1980
Pituitary	16	2070±2100	2790±2190	2320±2050	2970±2030
R. Hippocampus	13	2390±1770	4040±1780	2670±1700	4220±1570
L. Hippocampus	11	1800±1880	3770±2190	2040±1930	3850±2190
Hypothalamus	10	4290±1770	4920±1310	4390±1770	5040±1060

$D_{mean}$ = mean dose to the OAR; $D_{max}$ = maximum dose to the OAR.

The data in Fig. 3 are predominantly positive values, indicating the UN plans deliver more dose to the OAR than do the DN plans.^(13)^ The average median values of difference in $D_{\text{mean}}$ over all OARs is 170±90 cGy, while the interquartile range is 100±230 cGy. Similarly, the average difference in maximum values, $D_{\text{max}}$, is 140±140 cGy, while the interquartile range is 50±180 cGy. The Spearman's rank correlation rho between mean and max dose differences and OAR volume, CTV volume, patient age, and tumor location were calculated and no correlation was found.

Examining specific OARs, the median average dose increase to the eyes, temporal lobes, and pituitary in the UN plans are 0.70 (0.90 and 0.50 Gy for the right and left eyes, respectively), 1.7, and 1.8 Gy, respectively, being smaller for the eyes, which are situated further away from the treatment sites. Similarly, the right and left cochleas see a substantial increase in the median average for UN plans of 3.3 and 2.2 Gy, respectively. There is a similar increase in maximum dose for plans using the UN. The median maximum dose (over all patients) is 1.6 Gy larger for the spinal cord. Also, the median maximum doses to the right and left optic nerves are higher by 0.6 and 2.7 Gy, respectively. Finally, the median increase to the brainstem in UN plans is 2.5 Gy for the median average dose, and 0.5 Gy for the median maximum dose.

Changes to $D_{\text{min}}/D_{\text{Rx}}$ and $D_{\text{max}}/D_{\text{Rx}}$ from the robustness study are presented in Table 4. While the $D_{\text{mean}}/D_{\text{Rx}}$ values for CTV and OARs vary with perturbation values (±2 mm/±2.5%), the average of $D_{\text{mean}}/D_{\text{Rx}}$ over all perturbations does not vary significantly from the unperturbed values; hence, only the changes to $D_{\text{min}}/D_{\text{Rx}}$ and $D_{\text{max}}/D_{\text{Rx}}$ are presented in Table 4.

The robustness study indicates that changes to CTV DVHs are similar for UN and DN plans; the average $D_{\text{mean}}/D_{\text{Rx}}$ and $D_{\text{max}}/D_{\text{Rx}}$ for the CTVs changed by less than 1% for all plans, while the average $D_{\text{min}}/D_{\text{Rx}}$ decreased by 4% for both UN and DN plans. For the OARs, only the doses to the brainstem, optic nerves, and pituitary gland are presented in detail. While the mean doses to these organs, averaged over all patients and perturbations, remained unchanged with respect to the zero perturbation condition, the average $D_{\text{max}}/D_{\text{Rx}}$ tended to increase, between 0%–4%, for both UN and DN plans. The average $D_{\text{min}}/D_{\text{Rx}}$ of DN plans fell by 2%–6% more for the optic nerves and brainstem as compared to the UN plans, but no difference in $D_{\text{min}}/D_{\text{Rx}}$ of the pituitary gland was observed. These results are discussed further below.

**Table 4:** Change in prescription‐normalized minimum and maximum doses, $D_{min}/D_{Rx}$ and $D_{max}/D_{Rx}$, averaged over patients and perturbations to the CTV and OARs as a result of the robustness perturbations comprising ± 2 mm isocenter shifts with concomitant ± 2.5% CT Hounsfield unit and, hence, range variations

	*Dedicated Nozzle*		*Universal Nozzle*	
*Structure*	$△D_{\text{min}}/D_{\text{Rx}}$	$△D_{\text{max}}/D_{\text{Rx}}$	$△D_{\text{min}}/D_{\text{Rx}}$	$△D_{\text{max}}/D_{\text{Rx}}$
CTV	−4%	0%	−4%	0%
Brainstem	−11%	+4%	−5%	+3%
R. Optic Nerve	−6%	+1%	−2%	+2%
L. Optic Nerve	−4%	0%	−2%	0%
Pituitary	−9%	+2%	−10%	+2%

## IV. DISCUSSION

**Figure 4:**
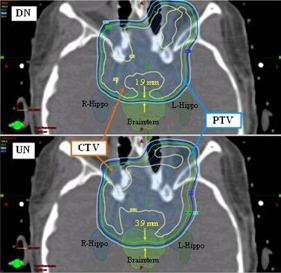
Dose distribution near the brainstem and hippocampus for plans made with the DN (top) and UN (bottom).

Statistical analysis demonstrates that the homogeneity index, HI, and inhomogeneity coefficient, IC, are not equivalent for DN and UN plans. Both metrics are smaller for DN plans, indicative of a more uniform dose distribution within the CTV. Outside the CTV, the dose gradient appears sharper for DN plans, which in most cases, leads to lower doses to OARs in proximity to the CTV. As an example, Fig. 4 shows the dose distribution around the CTV and PTV in the vicinity of the brainstem and hippocampus for plans that consist of equally weighted vertex and left lateral beams. The 95% isodose line (green) provides complete coverage of the CTV (brown) for both DN and UN plans, and the HI and IC values are similar for both plans: 10, 10% (DN, UN) and 4.3, 4.5% (DN, UN), respectively. Note, however, that the distance between the two arrows that mark the distance between the 85% and 95% isodose lines at the posterior edge of the PTV is 2 mm larger for the UN plan. As a consequence, the mean doses to the brainstem and hippocampus are approximately 700 cGy higher for the UN plan than for the DN plan.

In order to quantify the dose gradients outside target structures, the effective radii of 95% isodose and 85% isodose levels were calculated and the difference used as a measure of the strength of the dose gradients. Among the entire group of patients, the distance between the 95% and 85% isodose levels was found to be 0.5–1.0 mm larger in the UN plans than the DN plans for 12 patients; equal within ±0.4 mm for nine of the patients; and 0.8 mm smaller for just one patient. Thus, the trend of shallower dose gradients outside target volumes in UN plans than in DN plans follows for the majority of the UN plans, leading to higher mean and/or maximum doses in neighboring normal tissue structures.

The robustness study, summarized in Table 4, clearly indicates that the effect of ± 2 mm setup errors with concomitant ± 2.5% CT Hounsfield unit variation does not impact UN plans any differently than DN plans with respect to CTV coverage (i.e., minimal dose) and within 1% for the maximum OAR dose. Similarly, while no difference is observed in the minimum dose to the pituitary gland, DN plans tend to have a lower minimal dose for optic nerves and brainstem. Therefore the perturbations incurred by 2 mm/2.5% uncertainties are not counterproductive to the dosimetric advantage of the smaller DN spot profiles. We believe the differences in robustness of minimum and maximum doses to the same OAR, and minimum dose to different OARs, are related to dose gradients optimized to maintain target coverage at a higher dose when OARs are located proximal to target, enabling a sharp falloff when OARs are located further from target.

While the use of smaller spot spacing than specified in Eclipse TPS, noncoplanar treatment fields, and the number of treatment fields can impact plan quality, they also deviate from our standard treatment protocols and increase treatment time. To ensure a fair comparison between two nozzles, therefore, the impact of these parameters was not evaluated. A more judicious choice of beam angles would potentially benefit both UN and DN plans.

The overall trend in the data in Fig. 3 is that UN plans lead to higher OAR doses than DN plans; some structures (optic nerves/chiasm) may benefit more than others (pituitary gland) due to proximity to the target. However, it is observed that some OAR doses are found to be a bit higher (<500 cGy) in certain DN plans. In each of those instances, it was found that the OAR lies partially within the target structure. As the target coverage for DN plans is better than in UN plans with sharper penumbra beyond the treatment target, OARs overlapped with the treatment target will naturally have higher maximum and/or mean doses for DN plans. Both the HI and the IC are higher in UN plans, as a result of faster dose falloff at the target perimeter — a point that was discussed in the previous section.

Finally, while the reduction of mean and maximal dose may not be significant enough to impact vision or hearing loss, doses of this level can affect IQ development and the rate of secondary cancers;^(17)^ this is why proton therapy is felt to be particularly beneficial in the pediatric population.

## V. CONCLUSIONS

In a comparison of proton PBS plans for pediatric brain tumor patients using two distinct spot profiles from a UN and a DN, doses to normal tissue structures are higher for plans that utilize the UN beam with larger spots. As OAR structures in pediatric brain tumor patients are in close proximity to CTVs, as pediatric patients are particularly sensitive to developing late toxicities from radiotherapy to the brain. In light of the generally good prognosis and expected longevity of pediatric patients, it seems prudent for institutions with multiple PBS rooms to treat their pediatric patients in the room with the smaller set of sigma values for the advantage of reducing dose to OARs proximal to treatment target.
